# Case Report: Metreleptin Treatment in a Patient With a Novel Mutation for Familial Partial Lipodystrophy Type 3, Presenting With Uncontrolled Diabetes and Insulin Resistance

**DOI:** 10.3389/fendo.2021.684182

**Published:** 2021-06-08

**Authors:** Vaia Lambadiari, Aikaterini Kountouri, Eirini Maratou, Stavros Liatis, George D. Dimitriadis, Fredrik Karpe

**Affiliations:** ^1^ Second Department of Internal Medicine, Medical School, National and Kapodistrian University of Athens, Attikon University Hospital, Athens, Greece; ^2^ Department of Clinical Biochemistry, Medical School, National and Kapodistrian University of Athens, Attikon University Hospital, Athens, Greece; ^3^ First Department of Propaedeutic and Internal Medicine, Medical School, National and Kapodistrian University of Athens, Laiko General Hospital, Athens, Greece; ^4^ Medical School, Sector of Medicine, National and Kapodistrian University of Athens, Athens, Greece; ^5^ Oxford Centre for Diabetes, Endocrinology and Metabolism, Radcliffe Department of Medicine, University of Oxford and National Institute for Health Research (NIHR) Oxford Biomedical Research Centre (BRC), Oxford University Hospital Trusts, Oxford, United Kingdom

**Keywords:** diabetes mellitus, hypertriglyceridemia, metreleptin, familial partial lipodystrophy, PPARG, insulin resistance, case report

## Abstract

**Background:**

Familial partial lipodystrophy type 3 (FPLD3) is a very rare autosomal dominant genetic disorder which is caused by mutations in the peroxisome proliferator activated receptor gamma (*PPARG*) gene. It is characterized by a partial loss of adipose tissue leading to subnormal leptin secretion and metabolic complications. Metreleptin, a synthetic analogue of human leptin, is an effective treatment for generalized lipodystrophies, but the evidence for efficacy in patients with FPLD3 is scarce.

**Case Presentation:**

We present a 61-year-old woman, initially misdiagnosed as type 1 diabetes since the age of 29, with severe insulin resistance, who gradually displayed a more generalized form of lipoatrophy and extreme hypertriglyceridemia, hypertension and multiple manifestations of cardiovascular disease. She was found to carry a novel mutation leading to PPARG_Glu157Gly_ variant. After six months of metreleptin treatment, HbA1c decreased from 10 to 7.9% and fasting plasma triglycerides were dramatically reduced from 2.919 mg/dl to 198 mg/dl.

**Conclusions:**

This case highlights the importance of early recognition of FPLD syndromes otherwise frequently observed as difficult-to-classify and manages diabetes cases, in order to prevent cardiovascular complications. Metreleptin may be an effective treatment for FPLD3.

## Introduction

Lipodystrophies constitute a rare group of heterogenous genetic or acquired disorders which mainly characterized by partial or total loss of adipose tissue. Most often they are associated with cardiometabolic abnormalities including insulin resistance, diabetes mellitus, hypertriglyceridemia, non-alcoholic fatty liver disease, features of polycystic ovary syndrome (hirsutism, oligomenorrhoea, polycystic ovaries) and premature cardiovascular disease (1, 2). Akinci et al., in an international chart review study included 230 patients, observed the progression of lipodystrophy syndromes in patients who never received leptin or other lipodystrophy-specific treatment. Study results demonstrated that diabetes along with insulin resistant were present in 58.3% of patients and 82.6% of patients had triglyceride (TG) levels >150 mg/dl. Additionally, liver was the most common organ which displayed derangements and heart abnormalities were present in the 34% of patients with lipodystrophy (3). Lipodystrophies are classified into partial or generalized depending on the extent of fat loss. Further mechanistic classification can rely on genetic analysis using specific target genes for congenital generalized lipodystrophy or familial partial lipodystrophy (4, 5).

Familial partial lipodystrophy type 3 (FPLD3) is a rare type of lipodystrophy and results from mutations in the *PPARG* gene. Barroso et al. in 1999 described two different loss-of-function mutations in the ligand-binding domain of *PPARG* which were associated with diabetes mellitus, severe insulin resistance and hypertension (6). Later, Agarwal and Garg described the clinical phenotype of FPLD3 due to PPARG variants (7). FPLD3 is characterized by partial subcutaneous fat loss from upper and lower extremities, with preserved fat in the face, neck and truncal region (7). In contrast to other forms of FPLD, patients with *PPARG* mutation may display a mild lipodystrophy phenotype along with slightly decreased and even normal leptin levels. Indeed, a multicenter prospective observational study by Akinci et al., including data from 56 patients with FPLD, demonstrated that mutations in the *PPARG* gene are associated with less severe fat loss and higher levels of leptin compared to pathogenic variants in the *LMNA* gene (2). However, although the fat mass in the abdominal compartment is preserved, this may not necessarily mean the function of the tissue is normal (8). It is believed that the dysfunction of the subcutaneous fat depots is the underlying cause of ectopic fat deposition in liver and muscle (9, 10). Patients with FPLD3 usually present with metabolic disorders including diabetes mellitus, hypertriglyceridemia which can be severe in some cases (TG >500 mg/dl), subnormal leptin concentrations and signs of insulin resistance (acanthosis nigricans) (2, 11, 12).

Metreleptin, a synthetic analogue of human leptin that binds to and activates leptin receptor was firstly approved by U.S. Food and Drug Administration (FDA) in 2014 for patients with congenital or acquired generalized lipodystrophies (13). In 2018, metreleptin was approved by the European Medicines Agency (EMA) for adults and children aged ≥12 years with partial lipodystrophy (PLD) in whom metabolic control was not achieved with standard treatments (14). Since then, several studies have shown that metreleptin is an effective treatment for partial lipodystrophies (15, 16). However, the evidence regarding the efficacy of metreleptin in patients with FPLD3 is limited (17, 18). We are here presenting an FPLD3 successfully managed with metreleptin. A written informed consent for publication was obtained from the patient.

## Case Presentation

This is a 61-year-old woman who presented with diabetic symptoms and was misclassified as having type 1 diabetes with negative autoimmune-related type 1 diabetes antibodies (Tyrosine Phosphatase antibodies and Glutamic acid Decarboxylase antibodies). She was referred to our center due to uncontrolled diabetes despite the high doses of insulin and the coexistence of severe insulin resistance and decreased body mass index (BMI: 19.4 kg/m^2^). Age of presentation was 29 years and for the following 30 years she was treated with multiple daily insulin injections with high insulin requirements (5 IU/kg/day) together with metformin 2 g/day for 30 years, but constantly displaying poor glycemic control. Dyslipidemia was observed at the age of 35 years with raised cholesterol levels and severe hypertriglyceridemia despite intense lipid lowering therapy which lately consisted of the combination of fenofibrate 145 mg, rosuvastatin 40 mg/day and omega-3 fatty acids/day together with restricted fat intake. Hypertension was observed at the age of 5 years, and lately treated with the combination of nifedipine 120 mg/day, eplerenone 25 mg/day and hydrochlorothiazide 25 mg/day.

She also had micro- and macrovascular complications of diabetes indicating constantly insufficient glycemic control. She presented peripheral diabetic neuropathy (distal symmetric polyneuropathy) and nephropathy (eGFR: 43 ml/min, albuminuria: 314 mg/24 h). She also suffered from severe cardiovascular disease including peripheral artery disease and hypertrophic cardiomyopathy. Peripheral artery disease presented as intermittent claudication and carotid stenosis, for which she had a right carotid endarterectomy at the age of 52 years old. She was managed with clopidogrel 75 mg/day.

She had menarche at the age of 11, followed by irregular menstruation and then premature menopause at the age of 38. She also reported polycystic ovarian syndrome and never conceived. She underwent hysterectomy due to fibroids at the age of 54. She was diagnosed with osteoporosis at the age of 58 and has since received alendronate 70 mg/week.

### Family History

It has not been possible to conduct genetic family screening. Her mother and her maternal aunt displayed a lipodystrophy phenotype with fat loss of upper and lower extremities, presented diabetes from the ages of 25 and 28 respectively, and both showed early presentation of cardiovascular disease, presenting as ischemic stroke at the age of 45 years old and myocardial infarction at the same age, respectively. Her mother had reproductive problems and six miscarriages. Two out of four sisters and one brother developed hypertension and dyslipidemia at a young age.

### Genetic Analysis

A genetic analysis for lipodystrophy was performed and included the sequence and deletion/duplication analysis of the following genes: *ADRA2A*, *AGPAT2*, *AKT2*, *BSCL2*, *CAV1*, *CAVIN1*, *CIDEC*, *FBN1*, *KCNJ6*, *LIPE*, *LMNA*, *LMNB2*, *PCYT1A*, *PIK3R1*, *PLIN1*, *POLD1*, *PPARG*, *PSMB8*, and *ZMPSTE24*. The analysis revealed a novel heterozygous mutation in the *PPARG* gene (c470A>G, p. Glu157Gly, exon3) (The University of Chicago Genetic Services Laboratory). This particular amino acid change has not been described in other patients with *PPARG*-related disorders, but a different pathogenic sequence change affecting the same amino acid residue (p. Glu157Asp) has been described in a family with *PPARG*-related lipodystrophy (19). In our case the substitution of the negative charged glutamic acid at position 157 by the hydrophobic glycine could possibly lead to the formation of a non-functional protein responsible for the lipodystrophy phenotype. According to missense interpretation by experimental report classifier (MITER) the described mutation is associated with 78.6% probability of causing FPLD3 and 6.5-fold increased risk for type 2 diabetes mellitus.

### Examination

**Figure 1:**
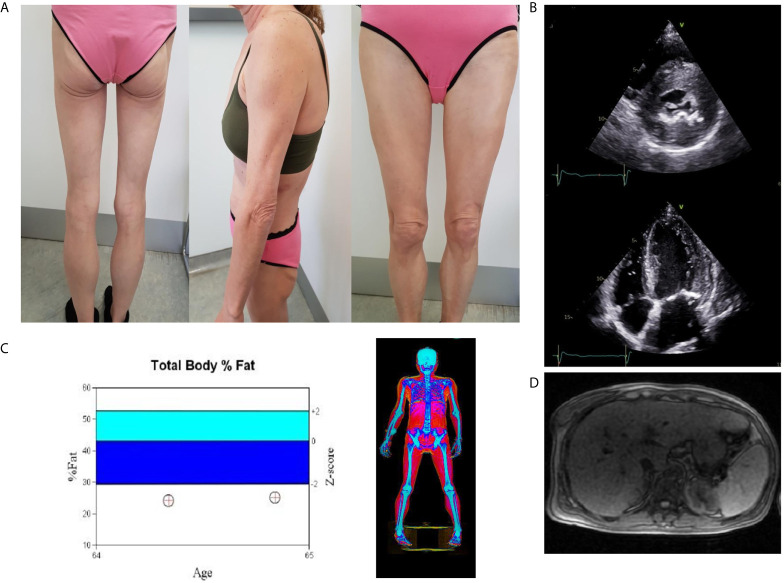
**(A)** Total lipoatrophy of extremities and gluteal area, **(B)** Two dimensional echocardiography revealed left ventricular hypertrophy (interventricular septum thickness 20 mm), **(C)** DXA showed increased fat tissue of the abdominal region and decreased fat mass of the upper and lower limbs. Follow-up DXA indicated unchanged total fat mass content, **(D)** The abdominal MRI image shows hepatic steatosis.

Examination revealed clinical signs of lipodystrophy of upper and lower limbs and gluteal area. Facial fat and abdominal prominence were also noted. She also had phlebectasia, hirsutism and cervical acanthosis nigricans. The patient mentioned that this phenotype was present from the age of 20 years old. Blood pressure was 175/84 mmHg.She was underweighted with a BMI: 19.40 kg/m^2^ (weight: 46 kg, height: 154 cm) (**Figure 1A**).

To assess the degree of regional fat loss in this case, we compared the total and proportional fat content in defined body regions using Dual-energy X-ray absorptiometry (DXA) with a group of healthy, lean postmenopausal women (20). The case has a relatively short stature and as a consequence a total lean mass not far from the lower end the 95% confidence interval of the comparators. The total fat mass was drastically lower giving a first indication of a lipodystrophic state. The visceral fat mass was similar to the comparators whilst the proportional fat content in extremities was drastically reduced showing the classic features of peripheral fat loss in partial lipodystrophy (**Table 1**).

**Table 1:** Regional body fat content in the case.

	Case	Healthy comparator group
		n = 13
**Age (years)**	64	59 (58–60)
**BMI (kg/m^2^)**	19.7	23.1 (22.7–23.5)
**Height (cm)**	153	161 (159–162)
**Total fat mass (kg)**	10.9	20.1 (19.2–21.1)
**Total Lean mass (kg)**	34.1	36.6 (35.5–37.6)
**Android subcutaneous fat mass (kg)**	0.6	1.0 (0.9–1.1)
**Android visceral fat mass (kg)**	0.3	0.3 (0.2–0.4)
**Gynoid fat mass (kg)**	1.3	4.0 (3.8–4.2)
**Arm fat%**	26.5	40 (38–41)
**Leg fat %**	18.7	40 (38–41)

Comparator values are mean (95% confidence interval).

### Biochemical Measurements

At the time of the first evaluation biochemical measurements revealed glycated hemoglobin (HbA1C) of 10%, a total cholesterol of 132 mg/dl of which high density lipoprotein (HDL-C) was 25 mg/dl. There was severe hypertriglyceridemia at 2.919 mg/dl. Further tests confirmed chronic kidney disease (blood urea nitrogen: 49.6 mg/dl, creatinine: 1.3 mg/dl, eGFR: 43 ml/min) and albuminuria (314 mg/24 h).

We performed glucagon stimulation test which revealed residual insulin secretion (fasting C-peptide: 2.09 ng/ml, 6 min after 1 mg of glucagon infusion C-peptide: 3.13 ng/ml). Plasma leptin concentration was close to zero (0.43 ng/ml).

### Imaging Assessment

Abdominal ultrasound confirmed hepatic steatosis and transient elastography (fibroscan) detected liver stiffness of 18 Kpa. Abdominal Magnetic Resonance Imaging (MRI) was unremarkable apart from hepatomegaly with a liver percentage fat fraction of 9.4%, and an enlarged spleen (**Figure 1D**). Cardiac MRI revealed left ventricular hypertrophy (maximal wall thickness 17 mm) and myocardial fibrosis in the basal interventricular septum (LGE SCORE 1/48, LGE mass 2%). Two-dimensional echocardiography revealed severe left ventricular hypertrophy, minimal mitral regurgitation, and ejection fraction of 60% (**Figure 1B**).

### Effect of Metreleptin Treatment

After noting her poor glycemic control, her antidiabetic treatment was intensified with empagliflozin 10 mg/daily and liraglutide 1.8 mg/daily, but this resulted only in a slight improvement in glycemic control after 3 months (HbA1c: 9.3%), despite additional strict dietetic management, but without any change in insulin management. We did not observe any change in triglyceride (TG) levels.

**Figure 2:**
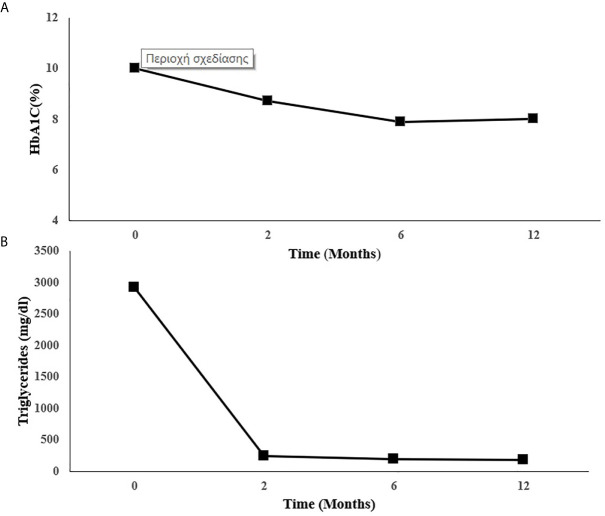
Changes in **(A)** HbA1C and **(B)** triglycerides during metreleptin treatment. HbA1C, Glycated hemoglobin.

Metreleptin was then initiated at 5 mg once daily on top of the current lipid and diabetes management. Glycemic control and hypertriglyceridemia improved within two months of treatment evidenced by decrease of HbA1C from 10 to 8.7% and the reduction of TG from a baseline value of 2.919 to 242 mg/dl. At six months there was further reduction in HbA1C (7.9%) and in TG (198 mg/dl). This improvement was sustained one year after treatment with metreleptin in the same dose (HbA1C: 8%, TG: 185 mg/dl-standard serum determinations are listed in **Table 2**), (**Figures 2A, B**). Insulin doses were reduced from more than 5 to 2.22 IU/kg/day. Blood pressure was also better controlled without any change in anti-hypertensive medication.

**Table 2:** Summary of the results over 12 months of metreleptin therapy.

	Baseline	2 months	6 months	12 months
**HbA1C (%)**	10	8.7	7.9	8
**TG (mg/dl)**	2.919	242	198	185
**Total cholesterol (mg/dl)**	132	137	115	106
**HDL (mg/dl)**	25	25	26	29
**LDL (mg/dl)**	–	64	49	46
**ALT (mg/dl)**	24	24	18	17
**AST (mg/dl)**	21	27	24	26
**BUN (mg/dl)**	49.6	56	25	39
**Creatinine (mg/dl)**	1.3	1.3	1.04	1.09
**Liver fat fraction (%)**	9.4	–	6.8	–
**Liver Stiffness (Kpa)**	18		18	
**BMI (kg/m^2^)**	19.40	–	–	18.98

ALT, alanine aminotransferase; AST, aspartate transaminase; BMI, Body Mass Index; BUN, blood urea nitrogen; HbA1C, glycated hemoglobin; HDL, high-density lipoprotein; LDL, low-density lipoprotein; TG, triglycerides. The biochemical assessment was performed after an 8-hour fasting.

At six months of metreleptin treatment a reduced liver fat content was observed (MRI estimated liver percentage fat fraction was reduced from 9.4 to 6.8%). However, liver stiffness assessed by transient elastography remained stable. A repeated DXA scan showed unchanged total fat mass content and cardiac MRI revealed no change (**Figure 1C**). Reduction in appetite following metreleptin treatment was reported. Hunger scales for the assessment of appetite were not used. Metreleptin treatment was well tolerated with no other adverse effects.

## Discussion

We present the case of a patient misdiagnosed as having poorly controlled type 1 diabetes for more than 30 years but finally identified as having FPLD3 with typical body clinical features and unusually low serum leptin concentration. Conventional and intensified antidiabetic treatment failed to normalize glycemic control but the introduction of metreleptin resulted in a drastic improvement of glycemic control and plasma triglycerides. Research data regarding the use of glucagon-like-peptide-1(GLP-1) agonists in patients with lipodystrophy are scarce and their effect has been described only in case reports (21, 22). Contrary to our case, in these previous reports authors pointed out that these regimens resulted in improvement of glycemic control and in reduction of insulin requirements, suggesting the positive effect of GLP-1 analogues in the management of diabetes in lipodystrophy syndromes (21, 22). It is noteworthy to mention that there is a concern regarding the use of GLP-1 analogues in patients with lipodystrophy and high TG levels. In previous cases, the triglycerides levels were lower (TG: 200–350 mg/dl) compared to our patient. However, the authors did not mention any adverse event (21, 22). Studies with more patients are required to evaluate the safety of these drugs in patients with lipodystrophy and severe hypertriglyceridemia. The risk of pancreatitis should be taken into account with the initiation of GLP-1 analogues in these patients. Metreleptin is an analogue of human leptin which imitates the physiological effects of endogenous leptin by binding to and activating the leptin receptor. Metreleptin treatment seems to ameliorate metabolic abnormalities including hypertriglyceridemia, hyperglycemia and insulin resistance. However, the mechanisms by which metreleptin improves these metabolic derangements are not fully elucidated. Leptin has a key role in the regulation of energy reserves and appetite by signaling the hypothalamus. In this regard, the reduction of hunger and calorie intake has been suggested as a mechanism responsible for the beneficial effect of leptin replacement (23–25). Püschel et al. showed that metreleptin treatment in four cases of FPLD3 improves satiety, reduces hunger and meal frequency (25). However, Oral et al. observed an additional favorable effect of leptin replacement on insulin sensitivity and on triglycerides metabolism that was independent of food intake (26). Additionally, Peterson et al. demonstrated that leptin treatment resulted in remarkable amelioration of insulin-dependent glucose metabolism which could be attributed to the improvement in insulin sensitivity in liver and muscle. These alterations are associated with a reduced hepatic and muscle triglyceride content (27). In accordance with these results, recently Brown et al. pointed out that metreleptin reduced peripheral and hepatic insulin resistance, decreased fasting glucose and triglycerides, and decreased liver fat content in patients with lipodystrophy whose food intake was held constant by a controlled diet (28). The preponderance of studies evaluating the efficacy of leptin treatment in patients with familial partial lipodystrophy included patients with lamin A/C (*LMNA*) pathogenic variants. However, the data regarding the effects of metreleptin in patients with FPLD3 are limited. So far, a few cases have been reported concerning the response of patients with *PPARG* pathogenic variant to metreleptin treatment, with controversial outcomes (16–18). Guettier et al. were the first who reported the efficacy of recombinant human leptin therapy in a case of a 36-year-old female patient with a heterozygous *PPARG* mutation. The patient presented a substantial amelioration in glycemic parameters along with a significant reduction in triglycerides after eighteen months of leptin treatment (18). Chong et al. conducted a prospective study to evaluate the effects of leptin replacement in 48 patients with different forms of lipodystrophy including two patients with mutation in *PPARG* genes. The results demonstrated that leptin replacement leads to significant and sustained amelioration of serum triglycerides and HbA1C (17). Recently, Sekizkardes et al. assessed the efficacy of metreleptin treatment in 22 patients with *LMNA* and in seven patients with *PPARG* pathogenic variants and highlighted that a 12-month metreleptin treatment led to significant reduction in HbA1C and insulin requirements. However, the efficacy of metreleptin treatment in terms of triglycerides levels was significant only in patients with *LMNA* pathogenic variants. The authors pointed out that the improvement in HbA1C and triglycerides levels following metreleptin treatment was more significant in patients with triglycerides >500 mg/dL or HbA1c >8% at baseline (16). According to the aforementioned research data, metreleptin treatment seems to improve glycemic control in patients with FPLD3. However, the data regarding the efficacy of metreleptin in hypertriglyceridemia in these groups of patients are inconsistent. Diker-Kohen et al., in a prospective open-label study, evaluated the efficacy of metreleptin in PLD compared to generalized lipodystrophy (GLD) and demonstrated that metreleptin treatment reduced HbA1C and triglycerides levels in GLD in a wider range of severity of baseline metabolic abnormalities compared to patients with PLD. Regarding PLD, patients with severe metabolic derangements and low endogenous leptin levels are more likely to respond to metreleptin treatment (29). In addition, the authors reported a significant reduction in antidiabetic and lipid-lowering medication and in total daily insulin in GLD patients after twelve months of metreleptin treatment. Interestingly, a decreased percentage of insulin use was also observed in GLD. However, only a trend for lower daily insulin requirements was observed in patients with PLD (29). In accordance with these results, Oral et al. demonstrated that improvements in metabolic parameters including HbA1C, fasting plasma glucose and triglycerides levels appeared to be less significant in PLD compared to GLD. In both PLD and GLD, higher levels of HbA1C and fasting TGs in baseline are associated with a greater response to metreleptin treatment (15, 30). Multi-society practice guidelines suggest metreleptin as a possible therapeutic option in patients with partial lipodystrophy who present with low levels of leptin (leptin <4 ng/ml) and severe metabolic abnormalities (HbA1c >8% and/or triglycerides >500 mg/dl) (31). Therefore, the degree of leptin deficiency and the severity of metabolic derangement may predict the efficacy of metreleptin in PLD, including FPLD3.The phenotypic heterogenity of lipodystrophy syndromes usually challenges the diagnosis between the different forms of lipodystrophy. Subcutaneous fat loss in FPLD is usually observed in extremities and gluteal area while the truncal fat is preserved (12). However, patients with partial lipodystrophy, like in our case, may possibly present with a more extensive fat loss which makes difficult the distinction between partial and generalized lipodystrophy (15). Furthermore, contrary to our case, PLD patients usually display slightly decreased leptin levels depending on the extent of fat loss, while in generalized lipodystrophy the widespread lack of adipose tissue results in low leptin levels (29). Thus, our case should raise awareness that patients with PLD may present with very low leptin levels and extensive fat loss. Due to the rarity of lipodystrophysyndromes, many general practitioners and medical specialists are not familiar with their diagnosis and their management and lipodystrophy disorders may frequently be unrecognized or misdiagnosed. Therefore, the delayed diagnosis in patients with lipodystrophies leads to the deterioration of quality of life and the increase of mortality and morbidity rates. Specifically, in patients with diabetes, the type of diabetes should be reassessed in cases of poor glycemic control despite the high doses of insulin, in conjunction with decreased body mass index and signs of insulin resistance. Furthermore, the suspicion is enhanced by the coexistence of negative antibody testing and residual insulin secretion in glucagon stimulation test. The case should: 1) raise awareness to clinicians of the diagnosis of lipodystrophy in patients with severe metabolic disorders despite intensified metabolic treatment, 2) affirm that patients with FPLD3 may benefit from metreleptin treatment, and 3) once again challenge the entity of difficult-to-manage diabetes patients.

## Data Availability Statement

The original contributions presented in the study are included in the article/supplementary material. Further inquiries can be directed to the corresponding author.

## Ethics Statement

Written informed consent was obtained from the individual(s) for the publication of any potentially identifiable images or data included in this article.

## Author Contributions

VL and SL were responsible for the diagnosis and treatment of the patient. AK, EM, and GD were responsible for the data collection and the search of the bibliography. VL and FK wrote and edited the manuscript. All authors contributed to the article and approved the submitted version.

## Conflict of Interest

The authors declare that the research was conducted in the absence of any commercial or financial relationships that could be construed as a potential conflict of interest.

## Abbreviations

BMI, Body mass index; DXA, Dual-energy X-ray absorptiometry; eGFR, Estimated Glomerular Filtration Rate; FPLD3, Familial partial lipodystrophy type 3; GLD, Generalized Lipodystrophy; GLP-1, Glucagon-like-peptide-1; HbA1C, Glycated hemoglobin; HDL, High density lipoprotein; LGE, Late gadolinium enhancement; LMNA, Lamin A/C; MRI, Magnetic Resonance Imaging; PLD, Partial Lipodystrophy; PPARG, Peroxisome proliferator activated receptor gamma; TG, Triglycerides.
